# ApoL1 risk allele accelerates high-fat diet-induced atherosclerosis in LDLR^−/−^ hamsters

**DOI:** 10.1016/j.gendis.2024.101379

**Published:** 2024-07-23

**Authors:** Yitong Xu, Wenxi Zhang, Jiabao Guo, Jinxuan Chen, Guolin Miao, Lianxin Zhang, Yufei Han, Jingxuan Chen, Ying Zhao, Xunde Xian

**Affiliations:** a Institute of Cardiovascular Sciences, State Key Laboratory of Vascular Homeostasis and Remodeling, School of Basic Medical Sciences, Peking University, Beijing 100191, China; b Department of Urology, China-Japan Friendship Hospital, Beijing 100029, China; c Beijing Key Laboratory of Cardiovascular Receptors Research, Peking University Third Hospital, Beijing 100191, China

Apolipoprotein L1 (ApoL1), coded by *ApoL1* gene that is only expressed in humans, gorillas, and green monkeys, is largely synthesized by the liver and incorporated into high-density lipoprotein (HDL), playing beneficial roles in inflammation and *Trypanosoma brucei* infection.^1^ To date, except for the neutral allele of ApoL1 G0 which is a major form discovered in humans, two human ApoL1 alleles have been identified, including ApoL1 G1 and ApoL1 G2, which are highly associated with various human kidney diseases in the population-based studies.^2^ Over the past two decades, the relationship between ApoL1 risk alleles and atherosclerotic cardiovascular disease remains largely unexplored. Different transgenic murine models expressing human ApoL1 risk alleles have been constructed; however, they are widely used to investigate the impact of ApoL1 on kidney diseases but not atherosclerosis because wild-type mice with overnutrient intervention are still resistant to atherosclerosis.^3^ Recently, emerging evidence shows that Syrian golden hamsters lacking low-density lipoprotein receptor (*LDLR*^*−/−*^) replicate familial hypercholesterolemia, providing an ideal animal tool for studying human atherosclerosis.^4^

It is worth noting that ApoL1 G1 has a mutation of amino acid at position 342 with or without the second mutation at position 384 to form ApoL1 G1^GM^ or G1^GI^, respectively, whereas ApoL1 G2 is termed two deletions both at position 388 and 389, making the haplotype frequency of ApoL1 G1 higher than ApoL1 G2. However, interestingly, increasing lines of evidence collected from human and experimental animal studies have shown that ApoL1 G1 and G2 equally contribute to kidney disease *in vitro* and *in vivo*, and given the complexity of ApoL1 G1, ApoL1 G2 allele transgenic mouse was largely developed to study the mechanism by which ApoL1 risk allele caused podocytopathy.^5^ Thus, in this study, it was rational for us to propose that ApoL1 G2 representing ApoL1 risk allele might play a key role in atherogenesis.

Based on the information from the GEO online database (GSE40231) using the patient-derived tissue samples from the atherosclerotic arterial walls and non-atherosclerotic arterial walls of patients with coronary artery disease, we found that the mRNA expression levels of ApoL1 were significantly elevated in atherosclerotic arterial walls, suggesting that ApoL1 may be a risk gene associated with atherosclerotic cardiovascular disease (Fig. 1A). However, this human data has not validated the causal effect of ApoL1 risk alleles on atherogenesis yet. Since liver is the major organ to produce ApoL1, we constructed a liver-specific adeno-associate virus 8 (AAV8) vector expressing ApoL1 G0 or ApoL1 G2 with AAV8-Null as a negative control to investigate the relationship between ApoL1 and atherosclerosis (Fig. 1A). Compared with AAV8-Null group, ApoL1 G0 and ApoL1 G2 proteins were successfully detected in circulation under 16-week chow diet condition without significant difference compared with human plasma samples (Fig. 1A), suggesting that ApoL1 expressed levels in *LDLR*^*−/−*^ hamsters are physiological and identical to the levels observed in humans. The hamsters expressing ApoL1 G2 displayed higher levels of plasma total cholesterol, HDL cholesterol, and non-HDL cholesterol relative to the AAV-Null or -ApoL1 G0 treated animals on a standard diet (Fig. 1B). However, ApoL1 G2 had no detrimental effects on the kidney, liver, and atherosclerotic progression (Fig. 1C). To our surprise, upon high-fat diet feeding, the plasma total cholesterol and non-HDL cholesterol levels were significantly reduced in both AAV8-ApoL1 G0 and -ApoL1 G2 treated groups when compared with AAV-Null treated controls, but there was no statistical difference between ApoL1 G0 and G2 groups (Fig. 1D). Interestingly, the hematoxylin/eosin staining and PAS (periodic acid-Schiff) staining revealed that only ApoL1 G0 expression caused more lipid accumulation and increased the glomerular and mesangial surface areas in the kidney (Fig. 1E). Moreover, although overexpressing ApoL1 G0 and ApoL1 G2 in the liver reduced the ORO (oil red O) positive areas, the latter led to enhanced inflammatory infiltration with an increase in apoptosis and CD68 (cluster of differentiation 68) without affecting fibrosis (Fig. 1E). Consistently, AAV8-ApoL1 G2 treated hamsters exhibited more atherosclerotic lesions in both whole aortas and aortic roots with more CD68 and MCP1 (monocyte chemoattractant protein 1), but less αSMA (alpha-smooth muscle actin) expression when compared with other two groups, suggesting that ApoL1 G2 could enhance local vascular inflammation to contribute to atherosclerotic development in a plasma lipid-independent manner in the setting of hyperlipidemia (Fig. 1F).

**Figure 1:**
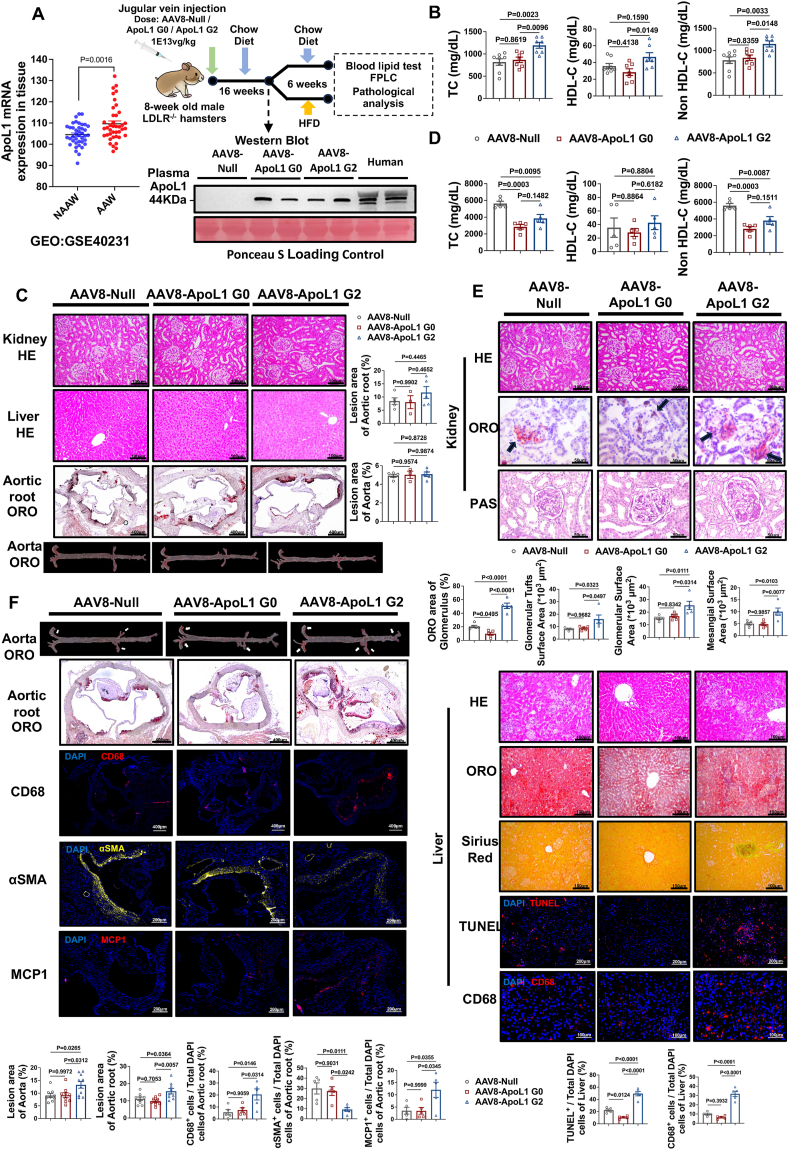
Detrimental effects of hepatic ApoL1 risk allele G2 expression on tissue homeostasis and atherosclerosis. (**A**) Left: Analysis of vascular ApoL1 mRNA expression in the GEO database. Top right: Schematic of the experimental design; Bottom right: Determination of plasma ApoL1 in *LDLR*^*−/−*^ hamsters injected with AAV8 expressing Null, ApoL1 G0, or ApoL1 G2; Ponceau S staining showed the plasma total protein. **(B)** Analysis of plasma TC, HDL-C, and non-HDL-C in AAV8-treated *LDLR*^*−/−*^ hamsters on a chow diet (*n* = 7 or 8). **(C)** Representative images of kidney and liver sections stained with HE, and of the aorta and aortic root stained with ORO and quantification in chow diet-fed *LDLR*^*−/−*^ hamsters (*n* = 3–5). **(D)** Measurement of plasma TC, HDL-C, and non-HDL-C in AAV-8 treated *LDLR*^*−/−*^ hamsters on HFD for 6 weeks (*n* = 5). **(E)** Top: Representative images of kidney sections stained with HE, ORO, and PAS and quantification in HFD-fed *LDLR*^*−/−*^ hamsters (*n* = 5); Bottom: Representative images of liver sections stained with HE, ORO, Sirius red, TUNEL, and CD68 in HFD-fed *LDLR*^*−/−*^ hamsters and quantification (*n* = 5). **(F)** Representative images of whole aorta and aortic root stained with ORO (*n* = 9 or 10), CD68, αSMA, and MCP1 (*n* = 5) and quantification in HFD-fed *LDLR*^*−/−*^ hamsters. The arrows indicate positive staining. All experimental procedures were approved by the Peking University Health Science Center (No. LA2010-059). The data were expressed as mean ± standard error of the mean and analyzed by one-way ANOVA/Tukey multiple comparisons test using Prism 9. AAV8, adeno-associate virus 8; ApoL1, apolipoprotein L1; αSMA, alpha-smooth muscle actin; HDL-C, high-density lipoprotein cholesterol; HE, hematoxylin/eosin; HFD, high-fat diet; CD68, cluster of differentiation 68; LDLR, low-density lipoprotein receptor; MCP1, monocyte chemoattractant protein 1; non-HDL-C, non-high-density lipoprotein cholesterol; ORO, oil red O; PAS, periodic acid-Schiff; TC, total cholesterol; TUNEL, terminal deoxynucleotidyl transferase dUTP nick-end labeling.

In summary, our present study reports for the first time that expressing human ApoL1 G2 *per se* in the livers of *LDLR*^*−/−*^ hamsters has no detrimental effect on atherosclerotic development; however, hepatic ApoL1 G2 expression coordinates with a second hit, such as high fat intake to cause severe hyperlipidemia, to exacerbate diet-induced atherosclerosis accompanied with kidney and liver injury. Therefore, our findings support the concept that ApoL1 risk alleles can increase the risk incidence of atherosclerotic cardiovascular disease in the context of hyperlipidemia, suggesting that atherosclerosis should be paid attention in ApoL1 risk allele carriers with hyperlipidemia and correction of ApoL1 risk alleles could be a potential therapeutic approach for the treatment of human atherosclerosis.

## Ethics declaration

All procedures were followed to the guidelines of Laboratory Animal Care (NIH publication no.85Y23, revised 1996), and the experimental protocol was approved by the Animal Care Committee, Peking University Health Science Center (LA2015-012).

## Conflict of interests

The authors declared no conflict of interests in this study.

## Funding

This work was supported by the 10.13039/501100001809National Natural Science Foundation of China (No. 82070460, 82270479, HY2021-1 to X. Xian), the Fundamental Research Funds for the Central Universities (China) (to X. Xian) and the Beijing Natural Science Foundation (No. 7242084 to X. Xian).

## Authors contribution

**Yitong Xu:** Data curation, Formal analysis, Investigation, Software, Validation, Visualization, Writing – original draft. **Wenxi Zhang:** Investigation, Validation. **Jiabao Guo:** Methodology, Resources. **Jinxuan Chen:** Methodology, Resources. **Guolin Miao:** Methodology, Software. **Lianxin Zhang:** Methodology, Resources. **Xunde Xian:** Conceptualization, Funding acquisition, Project administration, Supervision, Writing – original draft, Writing – review & editing. **Yufei Han:** Methodology, Resources. **Jingxuan Chen:** Methodology, Resources. **Ying Zhao:** Supervision.
