# Supplementary material for: ApoL1 risk allele accelerates high-fat diet-induced atherosclerosis in LDLR−/− hamsters
Source: Genes Dis. 2024 Jul 23;12(2):101379. doi: 10.1016/j.gendis.2024.101379 (PMC11635645; doi:10.1016/j.gendis.2024.101379)
Supplement: Multimedia component 1 [file mmc1.docx]

**Materials and Methods**

**Animals**

Golden Syrian hamsters were purchased from Vital River Laboratories (Beijing, China). Homozygous LDLR-deficient (LDLR^-/-^) hamsters were generated by CRISPR/ Cas9 genetic editing system in our lab as described previsouly^4^. For all experiments, all of the animals were matched for age and sex. Male animals were fed a chow diet (CD) (20% protein and 4% fat; Beijing Ke’ao Company, Beijing, China) or a high fat diet (HFD) containing 0.5% cholesterol and 20% fat with water ad libitum. AAV8-Null, AAV8-ApoL1 G0 and AAV8-ApoL1 G2 were injected into LDLR^-/-^ hamsters via the intrajugular vein at a dose of 1×10^13^ vg/kg. All procedures were followed to the guidelines of Laboratory Animal Care (NIH publication no.85Y23, revised 1996), and the experimental protocol was approved by the Animal Care Committee, Peking University Health Science Center (LA2015-012).

**Analysis of plasma ApoL1 and cholesterol**

Blood samples were collected from the retro-orbital plexus of the hamsters after 12-hour fasting under isoflurane anesthesia. 1 μL plasma from each animal was mixed with 4X SDS loading buffer (0.1 M Tris-HCl, pH 6.8, 2% SDS, 5% β-mercaptoethanol, 10% glycerol, and 0.05% bromophenol blue). The mixtures were boiled at 95°C for 10 min. Proteins were separated by 4-20% SDS-PAGE and transferred to a nitrocellulose membrane for Western blotting using antibody against ApoL1 (ab108315, Abcam, USA, Rabbit monoclonal IgG, 1:1000). Plasma total cholesterol (TC) levels were determined enzymatically using commercially available kits (Zhongsheng Beikong, Beijing). HDL-cholesterol (HDL-C) level was measured by the same TC kit after precipitating ApoB-containing lipoprotein by 20% polyethylene glycol (PEG). The value of nonHDL-C was calculated by the difference between TC and HDL-C.

**Pathological analysis**

To investigate atherosclerotic lesions and lipid accumulation in different tissues, all animals at the indicated endpoints of the experiments were perfused with cold phosphate buffered saline (PBS) and then fixed by 4% Paraformaldehyde (PFA). Whole aorta, heart, kidney and liver were harvested and embedded in OCT solution. 10 µm of frozen cross-sectioned slices of aortic roots and livers were used for morphological analysis. The atherosclerotic plaques in whole aortas (en-face) and cross-sectioned slices were visualized using 0.3% oil red O solution (Sigma-Aldrich, St. Louis, MO, USA). 3 μm of paraffin sections of livers were stained with HE and Sirius Red. 3 μm of paraffin sections of kidney were stained with periodic acid–Schiff (PAS). PAS staining without nuclear staining was used for morphometric analysis of the mesangial and glomerular surface areas was performed by pixel counts on a minimum of 50 randomly selected glomeruli per kidney section by ImageJ v.2.0 software (Image Processing and Analysis in Java)

For immunofluorescence staining, the stainings of CD68, αSMA and MCP1 in aortic roots and the stainings of CD68 and TUNEL in livers were analyzed using primary antibodies against CD68 (1:100 rabbit polyclonal IgG; BA3638, BOSTER) , α-SMA antibody (1:100 mouse polyclonal IgG; BM0002, Boster) and MCP-1 antibody (1:100 Rabbit polyclonal IgG;BA1843-2, Boster), respectively. The slices were then incubated with appropriate biotinylated second antibodies (1:1000, Donkey anti-mouse IgG (H+L) Cross-Adsorbed Secondary Antibody, Alexa Fluor 633；Donkey anti-rabbit IgG (H+L) Cross-Adsorbed Secondary Antibody,Alexa Fluor 555, Abcam)._­­_

**Statistical analysis**

All data were expressed as the mean ± SEM and evaluated using two-tailed Student’s t-test for two groups with one variable tested and equal variances, one-way ANOVA with Dunnett’s post-hoc or Tukey’s post-hoc for multiple groups w­ith only variable tested, or two-way ANOVA with Sidak’s post-hoc for plaque quantification. The differences were considered to be significant at p<0.05. Software used for data analysis was ImageJ (NIH) and Prism8.0 (GraphPad Software).
